# A Holistic Approach to Managing Microalgae for Biofuel Applications

**DOI:** 10.3390/ijms18010215

**Published:** 2017-01-22

**Authors:** Pau Loke Show, Malcolm S. Y. Tang, Dillirani Nagarajan, Tau Chuan Ling, Chien-Wei Ooi, Jo-Shu Chang

**Affiliations:** 1 Department of Chemical and Environmental Engineering, Faculty of Engineering, University of Nottingham Malaysia Campus, Jalan Broga, Semenyih 43500, Malaysia; showpauloke@gmail.com; 2 Institute of Biological Sciences, Faculty of Science, University of Malaya, Kuala Lumpur 50603, Malaysia; PauLoke.Show@nottingham.edu.my (M.S.Y.T.); tcling@um.edu.my (T.C.L.); 3 Department of Chemical Engineering, National Cheng Kung University, Tainan 701, Taiwan; dillirani@gmail.com; 4 Chemical Engineering Discipline and Advanced Engineering Platform, School of Engineering, Monash University Malaysia, Jalan Lagoon Selatan, Bandar Sunway 47500, Malaysia; ooi.chien.wei@monash.edu; 5 Research Center for Energy Technology and Strategy, National Cheng Kung University, Tainan 701, Taiwan

**Keywords:** microalgae, biofuel, biomass, lipid

## Abstract

Microalgae contribute up to 60% of the oxygen content in the Earth’s atmosphere by absorbing carbon dioxide and releasing oxygen during photosynthesis. Microalgae are abundantly available in the natural environment, thanks to their ability to survive and grow rapidly under harsh and inhospitable conditions. Microalgal cultivation is environmentally friendly because the microalgal biomass can be utilized for the productions of biofuels, food and feed supplements, pharmaceuticals, nutraceuticals, and cosmetics. The cultivation of microalgal also can complement approaches like carbon dioxide sequestration and bioremediation of wastewaters, thereby addressing the serious environmental concerns. This review focuses on the factors affecting microalgal cultures, techniques adapted to obtain high-density microalgal cultures in photobioreactors, and the conversion of microalgal biomass into biofuels. The applications of microalgae in carbon dioxide sequestration and phycoremediation of wastewater are also discussed.

## 1. Introduction

Energy is one of the basic necessities of modern life and is currently the most precious commodity required by consumers worldwide and by various industries. The generation of electricity for human consumption still relies heavily on fossil fuels such as petroleum, natural gas and coal [1]. In 2008, the annual energy consumed by the world was approximately 11,295 million tons of oil equivalent (Mtoe) [2]. The total primary energy supply (TPES) increased by almost 150% from 1971 to 2013, with a complete reliance on fossil fuels. More than 80% of the energy produced in the world comes in the form of fossil fuel [3]; nuclear and hydroelectric energies account for 5% and 6% of the total energy production, respectively [2]. The combustion of fossil fuel for electricity generation leads to many undesirable consequences such as pollution (as it accounts for a whopping 42% of the total CO_2_ emissions globally), depletion of non-renewable resources, and global warming [4]. One of the main products of fossil fuel burning, carbon dioxide (CO_2_), is a type of greenhouse gas that has a dire effect on the environment [5]. The global CO_2_ emissions increased from 22.7 billion tons in 1990 to 33.9 billion tons in 2011, despite 20 years of mitigation [6]. In 2006 alone, the CO_2_ emissions in the biosphere were estimated to be 29 Gtonnes [7]. The current atmospheric CO_2_ is 404 ppm, and it crossed 400 ppm in late 2013. According to a study conducted using the parallel ice sheet model by Winkelmann in 2015, the continual burning of fossil fuel is sufficient to eliminate the Antarctic ice sheet, causing a 58 meter global sea-level rise [8]. The vegetation in the Arctic region increased considerably from 1984 to 2012, owing to the increase in temperature, the changes in the annual growing season, soil physiology and soil nutrition [9]. Nature has its own way of absorbing excessive atmospheric CO_2_ via the plant’s photosynthesis and the oceanic carbon sink. However, these natural processes only remove about 12 Gtonnes of CO_2_ annually [10]. With the emergence of new economic superpowers such as China and India in the 21st century, the demand for energy will continue to rise and inflict more environmental deterioration [11]. In 1997, countries around the world signed the Kyoto Protocol, the agreement under which the industrialized countries will reduce their collective emissions of Greenhouse Gas (GHG) by 5.2% compared to the 1990 value [12]. The Paris Climate Conference in 2015 concluded with an agreement signed by 194 countries, to limit global the temperature increase to a minimum 2 °C, to peak greenhouse gas emissions early, and to arrive at alternative energy sources; in addition, the developed nations are to mobilize USD 100 billion a year to fund renewable energy research in developing countries. In order to protect the environment while meeting the increasing demand for electricity, it is imperative to seek out other types of renewable, environmentally friendly fuel sources.

**Figure 1:**
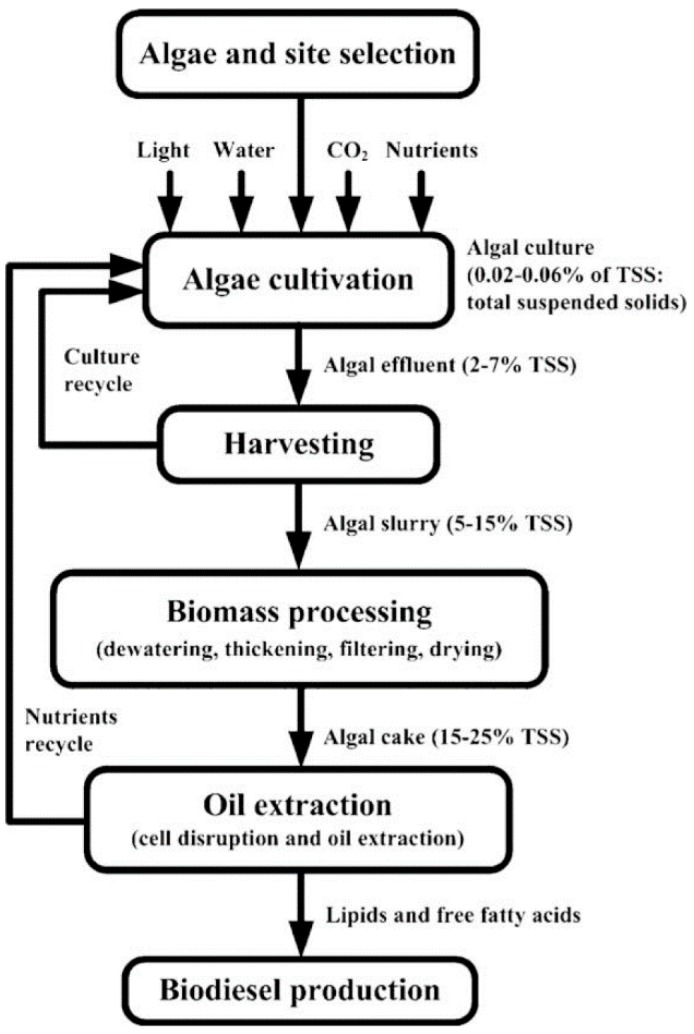
Stages of microalgae from cultivation to biofuel production. Used with permission from [14].

Biofuels can be derived from agricultural products such as starch, sugar, vegetable oil, and waste agricultural and lignocellulosic biomass, as shown in Table 1. The conflicts in land-use change and the competition of food crops with crop-based feedstock has led to the consideration of microalgal biomass (MAB) as a feedstock for biofuel production. Microalgae are rich in protein, lipids and carbohydrates, and the amount of each of these constituents can be tweaked to suit a specific need by the appropriate strain selection and the cultivation strategies [7]. The recovery of lipid from microalgae is one of the many efforts by scientists who are seeking out alternative fuel sources to break the monopoly of fossil fuel, and it has been studied extensively to maximize the extraction of lipids. The excellent performance of MAB in generating biofuel has increased the demand for the extraction of lipid from microalgae [13]. Methane, for instance, is one of the products of MAB. Methane is deemed as a highly desired fuel because of the availability of mature and stable technology for generating electricity from methane. Apart from that, the spent microalgae biomass (SMAB) can be reused to achieve large-scale production of biofuel; this increased the sustainability of algal biofuel by minimizing the microalgae waste. The growth of the alternative fuel sources is expected to increase, although their efficiencies could be limited by competition with other agricultural products for arable land, lack of infrastructure, high water and fertilizer requirements, and biodiversity conservation efforts [7]. A schematic diagram showing the different areas of discussion in this work is shown in Figure 1 [14].

This review provides a brief description of the current knowledge on the upstream and downstream processes in MAB management for biofuel application. This includes the cultivation of microalgae, the harvesting methodology, the biofuel conversion, and the application, challenges and future prospects of the microalgae biofuel industry. It is our hope that this review will be of value to all readers regardless of background in an attempt to propel the alternative fuel industry forward.

## 2. Microalgae as the Third Generation Feedstock

Microalgae are simple, unicellular or multicellular photosynthetic organisms and they utilize sunlight to fix atmospheric CO_2_ and convert it into biomass. Prokaryotic cyanobacteria, eukaryotic microalgae, and diatoms are among the most commonly studied microalgae for biofuel and fine chemical production in academia and industry [15]. For the purpose of this review, the term microalgae will include both prokaryotic and eukaryotic algae. Microalgae can be autotrophic or heterotrophic, based on their nutritional requirements and mode of growth. Autotrophic organisms perform photosynthesis as the main metabolic process using sunlight as an energy source and atmospheric CO_2_ as a carbon source. The cultivation of autotrophic microalgae requires a very simple nutritional requirement (e.g., mineral salts and vitamins), although the provision of sufficient illumination to the culture remains a challenge. The heterotrophic microalgae require an organic carbon source and can be grown devoid of illumination, because photosynthesis does not occur in this group of microalgae. The heterotrophic microalgae can be conveniently grown in a conventional bacterial bioreactor. Another category of microalgae is mixotrophic algae, i.e., algae that can undergo photosynthesis and can also assimilate exogenous organic carbon [16].

Autotrophic microalgae absorb light energy for growth. Only electromagnetic radiation in the visible light spectrum, ranging from 390 to 700 nm, can be absorbed by the light-capturing antennae associated with the photosynthetic machinery [17,18]. The net photosynthetic efficiency (PE) of the light-energy conversion, however, is quite low. The theoretical maximum PE of a green-type plant under sunlight was only 13% [18]. This figure represents the maximum possible PE achievable in theory, and disregards other factors that could reduce the efficiency, for instance insufficient light, cloudy weather, or photorespiration. Algae, on the other hand, can yield significantly a higher PE value due to their simpler structure [19,20,21,22,23].

### 2.1. Microalgal Cultivation for Biofuel Production

The research on the use of microalgae to generate biofuel began in the early 1960s, and was intensified in the 1970s during the first oil crises [24]. The United States then began a series of research programs aimed at studying microalgae as a renewable source of energy, which lasted from 1978 to 1996 [25]. The interest in generating biofuel from microalgae has recently been revived due to the volatility of the global crude oil price, along with the requirements by various governments to lessen GHG emissions from fossil fuel [14]. It is sustainable because microalgae can be grown year-round regardless of season and time, which gives it an advantage in terms of oil productivity compared to the best oilseed crops [26]. Moreover, microalgae require less water compared to other terrestrial crops, and can therefore reduce the cost of cultivation [27]. Another advantage of microalgae is that they can be grown in water unsuitable for human consumption or on non-arable land [28]. The selection of suitable algae species is one of the basic requirements to ensure a high quantity and quality of the biofuel output. Some algae are able to survive in the relatively harsh environmental conditions [29], as well as in the environment having a high level of CO_2_ [30]. Microalgae are rich in proteins, lipids and carbohydrates. Microalgae have been historically consumed as a source of protein to overcome dietary protein deficiency. Also, they are rich in several bioactive compounds such as antioxidant, anti-inflammatory, anti-angiogenic and anti-hypertensive. The direct consumption of dried microalgae as a probiotic, especially *Spirulina* sp. and *Chlorella* sp., has been recommended because of their nutritional values. As shown in Table 2, microalgae vary in their constituent lipid content, which can be extracted and converted to biodiesel. It is also possible to improve the lipid content by adjusting the growth parameters [31] such as CO_2_, light, and nutrients. The subsections below will discuss these growth factors in detail.

#### 2.1.1. Effect of Light Intensity on Microalgal Cultivation

As discussed above, microalgae can grow rapidly even under harsh and inhospitable environment. Nonetheless, in order to maximize the output of microalgae for biofuel application, the growth of microalgae must be monitored carefully. Given sufficient nutrients, sunlight, and CO_2_, most of the microalgae species can thrive. However, the diurnal cycles and seasonal variations may cause the availability of sunlight to change, therefore limiting the growth of microalgae [32]. The availability of sufficient light in the required intensity and spectral region (known as the photosynthetically active region, or PAR) of incident light is of prime importance for the cultivation of phototrophic microalgae. Light can affect the growth and metabolism of microalgae in three different conditions: light limitation, light saturation and light inhibition. Under light-limiting conditions, biomass growth and photosynthetic activity increase with the increase in light intensity. At light saturation, the rate of photon absorption exceeds the electron turnover, and therefore resulting in no further increase in the photosynthetic activity. With a further increase in the light intensity, an irreversible damage to the photosynthetic apparatus occurs, which is called photo-inhibition [33]. Below saturation, light is one of the key factors affecting the MAB productivity of autotrophic microalgae. Applying these principles to microalgal cultural techniques, it can be seen that light intensity cannot be maintained at the same level either in open ponds or photobioreactors (PBRs). For dense cultures or deep ponds, light inhibition or saturation can occur either at the surface of the culture or during light limitation/unavailabily. Microalgae can adapt to variations in light intensity by a process called photoacclimation/photoadaptation. Photoacclimation refers to the efficient utilization of available light by microalgae with a controlled set of responses mainly based on the redox status of the photosynthetic apparatus; photoacclimation covers a number of coordinated activities such as state transitions, thermal dissipation of excess light energy, differential expression of carotenoids to harvest light at the available maxima and changes in the light-harvesting complexes [34]. Having said all this, it is worth noting that the optimal light intensity for maximum biomass productivity varies according to the strain used. For example, the effect of light intensity on the growth of *Chlorella* sp. and *Nannochloropsis* sp. was studied at light intensities between 2000 and 10,000 lux. While *Chlorella* sp. achieved light saturation at 8000 lux, *Nannochloropsis* sp. showed an increase in biomass growth with an increase in light intensity up to 10,000 lux [35]. The isolate *Scenedesmus obliquus* CNW-N requires light at an intensity of 420 μmol/m^2^/s for maximum biomass production (840.56 mg/L/day), which was three-fold greater than the biomass produced by isolate grown at 60 μmol/m^2^/s with a light limitation occurred at 540 μmol/m^2^/s [36]. *Scenedesmus* sp. 11-1 showed a maximum growth at a light intensity of 400 μmol/m^2^/s with a biomass yield of 3.88 g/L, but there was no significant difference in the biomass between 200 and 400 μmol/m^2^/s and the biomass yield was 3.62 g/L at 200 μmol/m^2^/s. Even though the biomass yield did not vary, the maximum biomass was achieved in six days under high light condition, and in eight days under low light condition; this shown that the light intensity can affect the biomass productivity [37]. The *Neochloris oleoabundans* HK-129 biomass yield increased from 1.2–1.7 g/L when the light intensity was increased from 50 to 200 µmol/m^2^/s [38]. *Chlorella zofingiensis* was able to achieve 58% of its dry weight in lipid and 4.9 mg·g^−1^ dry weight in astaxanthin when exposed to high light and nitrogen deprivation conditions [39]. More information on the productivity of *C. zofingiensis* is shown in Table 3.

Sunlight can be used as the light source in outdoor cultivation, such as open pond systems; however, the depth of the ponds must be kept at a minimum of 20 cm to allow a maximum light penetration. Incident sunlight can also be used as a light source in outdoor PBRs; however, with the high cell densities achieved in PBRs, it is difficult to maintain an optimal light intensity throughout, as mentioned earlier. Since the 1990s, researchers have been using artificial lighting to counter this problem [40]. The usage of artificial lighting, however, could increase the production cost and thereby raising the price of end product. Moreover, the source of energy for the artificial lighting is also generated via fossil fuel, which could negate the positive impact of biofuel production. One possible solution to this problem is to understand the optimum absorption spectra for different algae species. By producing the specific type of spectra optimal to the growth of the algae, energy will not be wasted on generating unnecessary spectra, therefore reducing the energy demand. For instance, green algae contain chlorophylls *a* and *b*, and zeaxanthin, while diatoms generally contain chlorophylls *a* and *c*, and fucoxanthin [7].

#### 2.1.2. Effect of Temperature on Microalgal Cultivation

Temperature is one of the most important parameters for microalgal growth. Temperature affects microalgal growth the same way light intensity does: biomass production increases with an increase in the temperature up to the optimum temperature, whereby at above the optimum temperature, the growth of microalgae is inhibited. Below-optimal temperatures are not conducive for the efficient growth of microalgae because temperature affects the viscosity of the cytoplasm, the efficient utilization of nutrients and might also lead to photo-inhibition as the photosynthetic apparatus is not efficient. A lower temperature can also induce the accumulation of unsaturated fatty acids, which are used to overcome the problems of membrane fluidity. Growth inhibition at above-optimal temperatures is mainly attributed to heat stress that might denature functional proteins and photosynthetic enzymes [33]. A typical bell-shaped curve of microalgal growth can be obtained as a function of temperature, and the microalgal growth might vary with species and other environmental conditions. The maximum growth rates can be obtained at 17 °C for psychrophilic strains, 20–25 °C for mesophilic strains, and over 40 °C for thermophilic strains. In general, many common microalgae can thrive at temperatures between 15 and 30 °C, with an optimal growth rate at 20–25 °C [41]. Goncalvez et al. [42] studied the effect of temperature on biomass production and nutrient uptake on some common industrial microalgae such as *Chlorella vulgaris*, *Pseudokirchneriella subcapitata*, *Synechocystis salina* and *Microcystis aeruginosa*. It was observed that an increasing temperatures increased the biomass productivity, growth rates and efficient nutrient utilization; the optimal temperature for all the studied strains was 25 °C [42]. It must also be noted that the effect of temperature on growth or lipid accumulation varies with microalgae species. Among the green algae, species of the genus *Chlorella* vary widely in their optimal temperature, from a minimum temperature at 26 °C to a maximum temperature at 42 °C, reflecting the widespread ecological distribution of this genus [43]. When the growth temperature was raised from 20 °C to 25 °C, the lipid accumulation in *Nannochloropsis oculata* increased from 7.90% to 14.92%. Under the same conditions, an increase in culture temperatures from 25 °C to 30 °C decreased the lipid content of *Chlorella vulgaris* from 14.71% to 5.90% [44]. The optimal temperature for the growth of *Scenedesmus* sp. LX1 was found to be 20 °C, even though it can grow at temperatures between 10 and 30 °C. The increase or decrease in optimal temperature was accompanied by the physiological changes in the cell. When the temperature levels increased, the levels of polyunsaturated fatty acids in the cell decreased, while a decrease in the temperature brought about an increase in reactive oxygen species (ROS) in cells, indicating the presence of oxidative stress [45]. Most of the microalgae species can withstand temperatures as low as 15 °C, which is lower than their optimal temperature. At the other extreme, most of the microalgae species can not withstand temperatures much higher than their optimal level, at which a maximum increase of 2–4 °C higher than the optimal temperature can be tolerated [14].

An increase or decrease in temperature can induce the accumulation of carotenoids in microalgae, to overcome the oxidative stress induced by the altered temperature. An elevated temperature is a well-studied stress factor for the accumulation of astaxanthin in the marine alga *Haematococcus pluvilalis* [46,47]. Temperature is the only factor that could enhance the production of lutein [48]. Lutein accumulation has been associated with temperature stress in *Chlorella protothecoides* [49] and *Scenedesmus almeriensis* [50]. It was also suggested that temperature stress in combination with nutrient deprivation or light intensity variation could improve carotenoid accumulation.

#### 2.1.3. Effect of Nutrients on Microalgal Cultivation

The growth of microalgae for biofuel production is carried out in open or closed systems based on the applications of the biomass produced. The optimal nutrient provision during culture is of the utmost importance in attaining high-density cell cultures. The major nutrients required for the growth of microalgae include carbon, nitrogen, and phosphorus. Carbon accounts for about 40%–50% of the total cell content and it forms the basis for the central metabolism in the cells. The effect of CO_2_ source on photoautotrophic microalgal cultivation is discussed in the following section. Next to carbon, nitrogen is the second major nutrient, and it accounts for about 1%–10% of the total cell content as nitrogen is the major component in amino acids and nucleic acids. It is usually supplied as a nitrate, although other forms of nitrogen, such as ammonium and urea, can also be used. Some algae species can absorb nitrogen from the surrounding air in the form of NO_X_ [51,52], while other species require nitrogen in soluble form, i.e., urea [53]. Limiting the nitrogen intake of microalgae may also help enhance the lipid accumulation and slow the conversion of the free fatty acids in the lipid to triacylglycerol (TAG), which is the feedstock for biodiesel production processes [53]. Breuer et al. tested nine different microalgal strains including *Chlorella vulgaris*, *Chlorella zofingiensis*, *Nannochloris* sp. The Culture Collection of Algae at the University of Texas at Austin (UTEX) 1999, *Neochloris oleoabundans*, *Scenedesmus obliquus*, *Dunaliella tertiolecta*, *Isochrysis galbana*, *Phaeodactylum tricornutum*, and *Prophyridium cruentum*, for their growth properties and lipid accumulation in nitrogen-replete and -deplete conditions [54]. Among these, *Chlorella vulgaris*, *Chlorella zofingiensis*, *Neochloris oleoabundans*, and *Scenedesmus obliquus* accumulated a maximum lipid content of over 35% of their dry weight. The maximum productivity was achieved by *S. obliquus* UTEX 393 and *C. zofingiensis* UTEX B32, which was 322 and 243 mg/L/day, respectively; the biomass production of these two strains remained unaffected compared to the other strains, leading to a higher lipid productivity [55]. Nitrogen deficiency is also known to induce starch accumulation in microalgae. Under nitrogen-deplete condition for five days, *Chlorella zofingensis* attained a maximal starch productivity of 268 mg/L/day, with 66.9% by weight of carbohydrate content and 66.7% of which was starch [56]. Phosphorus is another major nutrient required for growth and metabolism in microalgae, as it is a constituent in energy transduction as well as the backbone of nucleic acids. Phosphates are always added in excess to culture medium because it has the tendency to form phosphate salts with the metals present in the culture. Microalgae are also capable of storing large amounts of phosphates as polyphosphate reserves to overcome any phosphate deficiency, and under phosphate-depleted conditions they secrete alkaline phosphatases to utilize any phosphates available in the environment. It was also shown that the phosphorus limitation can induce lipid accumulation in microalgae [57,58]. Other micronutrients including salts such as Fe, Mg, Ca, Na. K, Cl, and trace elements such as B, Cu, Mn, Zn, Mo, Co, V and Se, are necessary for cellular functions as some may act as a co-factor (Mg, Mn) while others are required in the biosynthesis process (Co, V). Essential vitamins are also added during cultivation and Si is required in the cultivation of diatoms [59]. The efficient illumination (in the case of phototrophic cultures), temperature, pH, and salinity need to be optimized based on the species to be cultivated.

The supply of nutrients to an algal culture is the most cost-incurring step in biofuel production. The strategy of nutrient recycling has been attempted previously in microalgal culture techniques. SMAB obtained after lipid extraction for biodiesel production was found to contain carbon and nitrogen which can be supplied as nutrients for microalgae, as a cost-cutting measure [60]. Pre-treatment is needed to break down the lipid and proteins in SMAB because these molecules are too complex to be utilized by microalgae. Rosch et al. studied the utilization of nitrogen and phosphorus by microalgae, and the results showed that a large amount of the nutrients sourced for microalgae growth can be recycled [61].

#### 2.1.4. Effect of CO_2_ Source on Microalgal Cultivation

The indispensable nutrient for microalgal cultivation is the carbon source; it accounts for more than 50% of the total biomass. For phototrophic microalgae, carbon is supplied in the form of CO_2_ gas. The CO_2_ used for microalgae growth can be derived from different sources: CO_2_ contained in the natural environment, CO_2_ discharged from anthropogenic industries, and CO_2_ from soluble carbonates [11]. The atmosphere currently contains 404 ppm of CO_2_, which can be directly absorbed by microalgae for growth. The partial pressure of CO_2_ in atmosphere is 0.04 Kpa and a minimum of 0.15 Kpa of CO_2_ is required to prevent a limitation of the kinetic uptake. Stoichiometrically, about 1.7–1.8 g of CO_2_ per gram of MAB is required and it can be as high as 3 g CO_2_ per gram of microalgae for the production of oil-rich algae [62]. The low levels of CO_2_ present in air are usually not sufficient enough to promote a higher biomass production. It must be noted that the major carbon-fixing enzyme, RuBisCo, has a very low affinity for CO_2_. In their natural habitats, microalgae overcome this insufficiency by adopting the carbon-concentrating mechanisms (CCMs). With CCMs, microalgae increase the intracellular CO_2_ concentration by active transport of inorganic carbon into the cells and the release of CO_2_ near RuBisCo by the activity of the carbonic anhydrase enzyme [63]. For commercial microalgal cultivation purposes, CO_2_ is supplied to the culture in gaseous form mixed with air, or as soluble inorganic carbonates such as Na_2_CO_3_ and NaHCO_3_. CO_2_ concentrations of about 1%–5% can often support a maximal microalgal growth, but generally laboratory algal cultures are aerated with 5%–15% CO_2_ routinely to overcome carbon limitation in fast-growing cultures [64]. *Chlorella pyrenoidosa* (FACHB 9) was tested for its ability to grow in ambient air and in the presence of CO_2_ from concentrations of 1%–20% [65]. The strain grew well over 1%–20% CO_2_ concentration, but the optimal biomass and lipid productivities (4.3 g/L and 107 mg/L/day, respectively) were achieved with 5% CO_2_. A decrease in CO_2_ concentration from 5% to 0.03% (air) increased the content of the saturated fatty acids and stimulated the expression of CCM genes similar to *Chalmydomonas reinhardtii* [65]. *Scenedesmus obliquus* SJTU-3 and *C. pyrenoidosa* SJTU-2 were cultivated with CO_2_ concentration up to 50%. An optimal growth of microalgae was observed at 10% CO_2_, and a higher concentration of 30%–50% CO_2_ supported the lipid accumulation in microalgae [66]. An increase in CO_2_ concentration could also increase the carbohydrate accumulation in some microalgae. A heat- and high light–tolerant microalga, *Acutodesmus* sp. was found to accumulate CO_2_ up to 40% of its dry weight when grown in 15% CO_2_, while a higher protein content of above 70% dry weight was obtained when grown in 5% CO_2_ [67]. Some microalgal species can accommodate a higher level of CO_2_ (as high as 150,000 ppm) [10,67]. This finding has prompted the feeding of CO_2_ from power plants [42,43,44,68,69,70,71] for microalgae cultivation. Otherwise, the carbon source can also be obtained in the form of soluble carbonates such as Na_2_CO_3_ or NaHCO_3_. Yeh et al. [72] tested the effect of sodium bicarbonate on the growth and biomass composition of *Chlorella vulgaris* ESP-31. The optimum biomass productivity was obtained at a concentration of 1.2 g/L, and a higher carbonate decreased the biomass concentration and the overall biomass productivity. When grown under optimal conditions, the biomass composition was 25%–30% of protein, 6%–10% of carbohydrate, and 30%–40% of lipid [72]. However, the use of sodium bicarbonate might increase the pH of medium, and therefore an alkali-tolerant microalga is a preferred choice. An extremely alkalihalophilic cyanobacteria, *Euhalothece* ZM001, was studied for its growth on bicarbonate as a carbon source using the approach of bicarbonate-based integrated carbon capture and algae production system (BICCAPS), which integrates the carbon capture and algal growth [73]. A maximum biomass productivity of 1.21 g/L/day was achieved with 1 M sodium carbonate/bicarbonate.

### 2.2. Microalgal Cultivation Methods: Open Systems and PBRs

Microalgal cultivation dates back to the 1950s during the Second World War when microalgae grown in open ponds were consumed as dietary supplements. Microalgal biotechnology and mass cultivation systems have come a long way since, and the closed systems have also been used successfully for the production of microalgal biomass in recent years. The two most significant methods for cultivating microalgae include: (1) open ponds; and (2) PBRs or closed systems. Each system has its own benefits and disadvantages. Research [30,57,74,75] shows that some cultivation methods can produce a higher yield than others, and some can be easily contaminated. In this section, we will briefly discuss each system in terms of its advantages and disadvantages. Moreover, different PBRs for microalgal cultivation will be explained and elaborated.

#### 2.2.1. Open Pond System

The open pond system is the oldest algal cultivation method and it was invented back in the 1950s [76]. As the name suggests, it requires a large body of water (lakes, ponds, or artificial ponds and containers) to operate. This system is capable of achieving high algae biomass production [77,78]. Open ponds can be natural or artificial, including natural lagoons, tanks, circular ponds and raceway ponds. Circular ponds are traditionally used for the cultivation of *Chlorella* sp. [79]. They are usually constructed in concrete and provided with a rotating arm to ensure mixing of the culture and to prevent sedimentation. Nowadays, raceway ponds are the most popular artificial algal cultivation system. It is basically a container designed to flow water in a loop, with a paddle wheel paddling the water in one direction. The paddlewheel is always in operation to prevent sedimentation and to ensure proper mixing of the nutrients in the culture. Raceway ponds are maintained at a depth of 20–50 cm to ensure the penetration of incident light, as the light intensity can limit the biomass production efficiency. In comparison to PBRs, raceway ponds are cheaper, suitable for locations with marginal crop production potential [79], low in energy requirement [57] and maintenance [80], and highly profitable [57]. Nonetheless, due to the continuous looping of water in an open pond system, it is also more prone to contamination and invasion by other algae species and protozoa [32]. The solution to this problem is to create an extremely specific environment suitable only for a certain type of algae species, for instance the *Dunaliella salina* is adaptable to a very high salinity, and *Spirulina* spp. is adaptable to a high alkalinity [76]. This approach, however, is unable to prevent contamination from bacteria or other biological contaminants [81]. Other major drawbacks of open pond systems include the influence of seasonal variations, evaporational water losses, poor light utilization efficiency, diffusion of the inlet CO_2_ into the atmosphere and large land requirements [82]. Table 4 shows the biomass productivity figures for open pond production systems.

#### 2.2.2. PBRs

PBRs are bioreactors used for the cultivation of microalgae and cyanobacteria. They provide a closed environment for the cultivation of microalgae with a provision for efficient capture and utilization of light. PBRs can be operated indoor or outdoor; the indoor PBRs use artificial light sources to illuminate the culture. Microalgal cultures in PBRs are not in direct contact with their environment, which prevents contamination issues and axenic culture maintenance [83]. Gas transfer and exit are provided by a filtered gas exchanger, preventing any direct contact with air. They also provide a better control over the culture conditions used, such as pH, temperature, light, salinity and CO_2_ concentration. The use of a particular design of PBR for the microalgal culture depends heavily on the species to be cultivated and the nature of the end product [84]. Despite the high cost involved in the installation and maintenance of PBRs, they are the reactors of choice for production of high quality pharmaceuticals and health supplements from microalgae, such as pigments or fatty acids. Factors to be considered for the design of a PBR are as follows [85,86]: they should be of versatile design and allow the cultivation of various microalgal strains; they must provide uniform illumination of the culture and assist in efficient transfer of CO_2_ and O_2_; they should support minimum fouling of the culture vessel used; the design should provide for minimum evaporational water loss and CO_2_ loss; the design should incorporate a high surface-area-to-volume ratio for efficient illumination and should have minimum non-illuminated or dark zones; the design should support cultures with high rate of foaming; and high rates of mass transfer should be maintained for achieving high-density cultures.

Based on the reactor design, PBRs can be classified into stirred-tank PBRs, vertical column PBRs, tubular PBRs, flat panel PBRs and so on. A hybrid PBRs combine one or two of the above designs to complement each other and to overcome the disadvantages of one particular design of PBR. Conventional bacterial fermenters, also called fermenter-type reactors, are being used for the cultivation of heterotrophic microalgae as well.

##### Stirred-Tank PBRs

Stirred-tank PBRs are the conventionally used bioreactors for the cultivation of industrially important bacterial cultures. Agitation is provided by means of impellar blades of different sizes and shapes, and the vortex is reduced by the installation of baffles. Aeration is provided via bubbling of CO_2_-enriched air from the bottom of the vessel with an air sparger [87]. For the purpose of microalgae cultivation, this type of bioreactor has been modified to have external illumination of the culture vessel by means of fluorescent lights or optical fibers. The presence of a large disengagement zone in this stirred-tank PBRs aids in the separation and the release of unused CO_2_ and photosynthetic O_2_ [87]. Stirred-tank PBRs offer a better control over the process parameters and the maintainance of culture’s sterility. However, because of the lower surface-area–to-volume ratio, incident light is not utilized efficiently by the culture, thereby reducing the photosynthetic efficiency. Ogbonna et al. designed a novel internally illuminated stirred-tank PBR for the cultivation of *Chlorella pyrenoidosa* and they found that the new internally illuminated stirred-tank PBR was highly efficient for high-density cell cultures because the light intensity ensured by the uniform distribution of the light in the reactor was three-fold higher than any commercially available PBR [88].

##### Vertical Column PBRs

Vertical column PBRs use transparent vertical columns for the cultivation of microalgae. They are usually cylinders with radii of up to 0.2 m and a height of 4 m or less. The small radii increase the surface-area-to-volume ratio and the length is determined by the gas transfer limitations [86]. Vertical column PBRs are characterized by high surface-to-volume ratios, user-friendly operations, compact size and high MAB production. Gas is supplied with spargers from the bottom of the cylinder. The gas is provided as small bubbles, which also provide gentle mixing of the culture without causing any shear stress [83]. The aeration rate should be maintained at an optimum rate; less aeration equals to less agitation, causing cells to be stagnant in dark zones, and an increase in aeration rate causes the accumulation of microbubbles, affecting light penetration [89]. Based on the air flow pattern, vertical column PBRs can be categorized into bubble column or air-lift PBRs [86].

Bubble column PBRs are cylindrical PBRs with a height of at least twice their diameter [90]. An external light source is used to illuminate the culture, and the gas is sparged from the bottom of the cylinder by means of a sparger. Since the gas released provides the required mixing and gas transfer, the design of the sparger is crucial for the design of a bubble column PBR. Bubble column PBRs are advantageous because of their low capital cost, high surface-area-to-volume ratio, lack of moving parts, satisfactory heat and mass transfer, relatively homogenous culture environment, and efficient release of O_2_ and residual gas mixture [91]. Since mixing is provided by general turbulence, improper mixing can lead to a reduced photosynthetic efficiency as cells might stay longer in the interior dark zone. Turbulence can be increased by installing perforated plates which might be required during scale-up.

Air-lift PBRs are the most efficient type of PBRs because of their efficient mixing properties. The design consists of two interconnecting tubes: air is sparged from the bottom and is distributed in the “gas riser”, causing turbulence and liquid flow; the second column, called the “downcomer”, receives the liquid flow from the gas riser, and eventually the liquid is recirculated between the riser and downcomer. The liquid flow creates a circular and homogeneous distribution of cells causing flashing light effects that increase productivity [86,92]. Air-lift PBRs are generally recommended for fragile microalgae which are highly sensitive to shear stress, and a high biomass productivity can be achieved with an efficient mixing in the reactor [90]. Based on the liquid flow, air-lift PBRs can be categorized into internal loop air-lift PBRs, external loop air-lift PBRs and split column air-lift PBRs. Of these, the external loop configuration ensures a better mixing because of the distance between the riser and the downcomer, which enables an efficient gas disengagement.

##### Horizontal Tubular PBRs

Tubular PBRs are most commonly used for the commercial production of microalgae, and it differ from the vertical column PBRs in terms of surface-area-to-volume ratio, gas dispersion ratio, mass transfer characteristics, fluid movement and illumination levels [86]. Tubular PBRs are typically composed of transparent polypropylene acrylic or polyvinylchloride pipes with small internal diameters arranged in different configurations, such as horizontally, helically, vertically, inclined, α-type and so on [33]. A minimum diameter of the tube should be maintained to ensure an efficient light penetration; an increase in the diameter of tube will decrease the surface-area-to-volume ratio, thereby affecting light capture. The length of the tube should also be optimized, as an increase in the length of the tubular PBR may cause oxygen hold-up and CO_2_ starvation between the gas exchange units. A width between 10 and 60 mm is optimal, while the length of tube may vary, keeping the surface-area-to-volume ratio above 100/m, which is the characteristic of this design [93]. Mixing and agitation of the culture are maintained by an air pump to provide circulation, and gas transfer to the culture may vary from low to high, depending on the flow characteristics and the air-supply technique adopted. Among the various configurations, coil-shaped or helical tubular PBRs are often chosen due to their efficient use of space.

##### Flat Panel PBRs

As the name suggests, flat panel PBRs use flat panels as the culture vessel rather than a column or a tube. The flat reactor design enhances light penetration and aids the culture in achieving the maximum photosynthetic efficiencies. A high surface-area-to-volume ratio, open gas disengagement systems, the distinct inclination of the channels to receive optimal light and the absence of mechanical devices are the hallmark features of flat panel PBRs [86]. Aeration and agitation are achieved by bubbling air from the base of each panel (air-lift) or by rotation using a motor (pump-driven). The temperature is maintained by water spray or internal heat exchangers, and the oxygen holdup is comparatively low in flat panel PBRs. The transparent material used for the PBR is usually made of glass or polycarbonate, which allow the maximum light penetration [90]. The thickness of the material influences the surface-area-to-volume ratio and the length of the light path, therefore it should be optimal. Although thin panels are preferred in terms of efficient light penetration and increased biomass productivity, they are extremely difficult to make, and they are more prone to light inhibition of algal cultures, temperature fluctuations and fouling. The use of flat panel PBRs is limited by the large land requirement, the elaborate setup with many units, fouling of the reactors, temperature fluctuations and light inhibition in outdoor cultures, sterilization issues and damage associated with aeration [90].

#### 2.2.3. Hybrid System

A PBR combined with an open pond system will result in a hybrid two-stage cultivation system. The PBR is the first stage, consisting of controlled conditions where microalgae can grow with a reduced risk of contamination, followed by the second stage where the cells will be exposed to nutrient stresses to improve the production of the desired lipid [73,93] in an open pond system. The effectiveness of this two-stage system has been tested using microalgae *Haematococcus pluvialis*, and it achieved an annual mean oil production rate of more than 10 toe·ha^−1^ per year [94]. Using the similar growth conditions, other microalgae species with a higher lipid content could possibly achieve rates as high as 76 toe·ha^−1^ per year.

#### 2.2.4. Heterotrophic (Fermenter) System

Some of the strains of microalgae have the ability to grow not just under phototrophic conditions, but also to utilize organic carbon under light-free conditions. For this production, microalgae are cultivated on organic carbon substrates free from light sources [95], for example fermenters or glucose in stirred-tank bioreactors. The heterotrophic system allows a flexible growth control while reducing the harvesting cost, thanks to the higher cell densities achieved [96]. Moreover, the set-up cost is also comparatively lower, in spite of the higher amount of energy required for the initial production of organic carbon sources [82]. Numerous authors [75,97,98,99] have studied, using multiple types of microalgae species (*Chlorella protothecoides*, *Galdieria sulphuraria*, *Crypthecodinium cohnii*), the large-scale production of biodiesel using this system; for the species *C. protothecoides*, the lipid content in the heterotrophic cells could be four times higher than that of autotrophic cells under similar conditions.

#### 2.2.5. Mixotrophic System

Some algae species are able to perform photosynthesis and also metabolize organic carbon sources such as glucose [100,101]. For these mixotrophs, the light source is not an indispensable requirement for growth [102], because in the absence of light, these organisms can utilize organic carbon sources released as CO_2_ by themselves from the respiration process for growth [100]. This lowers the effect of biomass loss during the dark period of the diurnal cycle [102]. Nonetheless, Chojnacka and Noworyta [103] found that mixotrophic culture has lowered photoinhibition and enhanced growth rates compared to autotrophic and heterotrophic cultures.

### 2.3. Potential Applications of Microalgae

#### 2.3.1. CO_2_ Sequestration

Flue gases from power plants make up more than 7% of the total world CO_2_ emissions [104], while industrial exhaust gases make up 15% [105,106]. Microalgae can absorb CO_2_ from several main sources: the CO_2_ in the atmosphere, the CO_2_ released from power plants, and the CO_2_ from soluble carbonate [12]. The absorption of CO_2_ from air by microalgae is the most fundamental form of carbon capture. However, the low concentration of CO_2_ content (about 0.04%) in the atmosphere renders the large-scale microalgae cultivation infeasible [107]. Flue gas typically consists of 9.5%–16.5% (*v*/*v*) CO_2_, 2%–6.5% (*v*/*v*) O_2_, 100–300 ppm NO_x_, 280–320 ppm SO_x_, CO, heavy metals and particulate matter [108]. The high CO_2_ in flue gases may be directly supplied to the microalgae culture as a supplement to the inorganic carbon requirements of high-rate algal cultures, which are otherwise aerated by commercial CO_2_ gas. Even though CO_2_ is abundant in other sources, the commercially pure CO_2_ is expensive. Around 1.83 kg of CO_2_ can be absorbed for every kg of algal biomass [82], though the CO_2_ sequestering ability of each microalgae differs (Table 5). Unfortunately, the presence of other toxic gases such as SO_x_ and O_x_ can only be tolerated by certain types of algae. An ideal microalgal strain that can use the exhaust flue gas directly should possess the following characteristics: (i) able to tolerate high concentrations of CO_2_; (ii) able to grow at high temperature as the temperature of the exhaust gas is around 150 °C, and even after primary cooling, a temperature of about 50 °C can be expected; (iii) able to tolerate the other toxic compounds present in the flue gas such as SO_x_ and NO_x_, CO, etc.; (iv) possesses a high biomass productivity as the resultant biomass would be further used for biofuels production; (v) able to tolerate nutrient limitations and fluctuations in pH in case of continuous flue gas feeding. Microalgae species *Scenedesmus dimorphus*, *Botryococcus braunii*, *Chlorella vulgaris*, and *Nannochloropsis oculate* are the most promising species for CO_2_ sequestration [109]. *Chlorella pyrenoidosa* PY-ZU1 was mutated by nuclear irradiation and domesticated gradually with increasing concentrations of CO_2_ and with the optimal light intensities and mixing, and a CO_2_ fixation rate and efficiency of 1.54 g/L/day and 32.7% were obtained [110]. *Chlorella* sp. H-84, *Chlorella* sp. KR-1 and *Chlorella* sp. ZY-1, as well as *Chlorella* sp. T-1, can grow in the presence of a 40%, 70%, and 100% of CO_2_ supply, respectively [111]. *Chlorella* sp. MTF-15, a more thermo-tolerant species developed by mutagenesis, was cultivated using flue gas from steel plants in outdoor vertical column PBRs. A maximum growth rate and lipid production of 0.762/day and 0.961 g/L, respectively, were achieved [112]. Similarly, *Scenedesmus obliquus* strain WUST4 obtained by UV mutagenesis was able to tolerate and grow in the presence of high CO_2_ concentration and it has a high carbon fixation efficiency. When grown with actual flue gas in an air-lift PBR, a CO_2_ removal rate of 67% was achieved [113]. The implementation of microalgae to sequester CO_2_ from power plants has so far yielded positive results.

#### 2.3.2. Wastewater Treatment

Other than that, microalgae are also used for the dual role of phycoremediation of domestic wastewater [114]. Wastewater generated from various industries and domestic use is rich in nutrients such as carbon, nitrogen, phosphorus and dissolved oxygen. When discharged into the environment without proper treatment, this nutrient-rich wastewater can lead to eutrophication and harmful algal blooms in natural environments, low dissolved oxygen concentrations, fish deaths, undesirable pH shifts, and cyanotoxin production. Chemical treatments can also be done for nutrient removal but the removal of secondary sludge is another environmental issue. Microalgae can be conveniently grown in wastewater as they are rich in nutrients required by microalgal cultivation, ultimately resulting in the efficient removal of nitrogen, phosphorus and dissolved oxygen [115]. The high-rate algal ponds have been traditionally used for both the treatment of wastewater and the simultaneous production of algal biomass. The most commonly found algae in wastewater ponds include some species of *Chlorella*, *Scenedesmus*, *Micractinium*, *Euglena*, and *Chlamydomonas*; *Oscillatoria* may be found in ponds with excessive loadings or long residence times [116]. The illumination of the microalgal cultures in wastewater could pose a problem, depending on the water quality. The cultures could be carbon-limiting as wastewaters are deficient in inorganic carbon. An efficient illumination of the culture combined with CO_2_ aeration, after certain pre-treatment processes to remove suspended solids for improving water quality, might result in maximal biomass production associated with wastewater treatment [117]. Some researchers have studied the use of heterotrophic algae which can utilize organic carbon in the absence of light for the treatment of wastewater. Zhang et al. tested *Scenedesmus* sp. and *Chlorella* sp. for heterotrophic growth in domestic wastewater under dark condition. The biomass content of *Scenedesmus* sp. ZTY3 and *Chlorella* sp. ZTY4 increased by 203% and 60.5%, respectively, compared to the initial cell densities and the lipid contents, which were 55.3% and 79.2%, respectively. The efficiencies of nutrient removal were also high, i.e., 52.9% for *Scenedesmus* sp. ZTY3 and 64.4% for *Chlorella* sp. ZTY4 [118]. Wastewater remediation using microalgae is an eco-friendly process [119] with no secondary pollution, as long as the biomass produced is re-used to allow an efficient nutrient recycling. By using microalgae to absorb the chemical and the organic contaminants as nutrients, the MAB produced for biofuels not only can save the cost in term of fertilizer, but also treat wastewater [117]. Chinnasamy et al. evaluated the efficiencies of 13 strains of microalgae in removing nutrient from carpet industry wastewater containing 85%–90% carpet industry effluents and 10%–15% municipal sewage. Among the genera studied, *Scenedesmus* seemed to be dominating in the consortium and 96% removal of nutrients was obtained. Using treated wastewater in raceway ponds, a biomass production potential of 9.2–17.8 tons·ha^−1^ per year could be achieved. The lipids extracted from the biomass was found suitable for biodiesel production [120].

#### 2.3.3. Other Potential Applications of Microalgae—Food, Animal Feed, Cosmetics and Fertilizer

The application of microalgae as food is limited due to current food safety regulations [121], but it has been used historically as a dietary supplement. Several common species of microalgae used in the food industry are some entities of the genera *Chlorella*, *Spirulina* and *Dunaliella*. These strains are usually sold as health supplements or as food additives. *Spirulina* sp., for instance, is a popular health supplement that can boost the immune system and prevent viral infections [122]. The protein content of *Spirulina* sp. is very high (up to 75% by dry weight) and it contains all the essential amino acids such as valine, leucine and isoleucine. The protein from microalgae is comparable to or even better than those of wheat and other vegetables, and also they are characterized by high digestibility [122]. They have an annual production reaching 3000 tons in dry weight, and are produced in various countries such as China, Taiwan, India, Myanmar and Japan [24]. Seaweeds or edible marine algae are rich in sulphated polysaccharides with a host of pharmaceutical applications. Sulfated polysaccharides include carrageenan from red algae, ulvan from green algae, along with laminarin and fucoidan from brown algae. Polysaccharides of marine algae are known to possess anti-coagulative, anti-viral, anti-lipogenic, anti-carcinogenic and immune-modulating activities [123]. Several types of antioxidants—compounds with the ability to fight aging—have been successfully segregated from microalgae [14]. The pigments of microalgae, i.e., chlorophyll and carotenoids, which are essential for photosynthesis and oxidative stress maintenance, can be extracted and used as antioxidative supplements. Microalgae of the genera *Spirulina*, *Botryococcus*, *Chlorella*, *Dunaliella*, *Haematococcus* and *Nostoc* have been recognized as potential sources for microalgal pigments that can be used as nutraceuticals. *Dunaliella salina* was the first commercially cultivated microalgae for the production of β-carotene. *Haematococcus pluvialis* is cultivated in open ponds and raceways for astaxanthin production. *Murellopsis* sp., *Scenedesmus almeriensis*, and *Chlorella protothecoides* are the known producers of lutein, i.e., a pigment very promising in the alleviation of age-related macular degeneration [33]. The biological activities of microalgal pigments include antioxidant, anti-carcinogenic, anti-inflammatory, anti-obesity, anti-angiogenic, and neuroprotective [124]. Microalgae are also viewed as a sustainable source for polyunsaturated fatty acids, such as eicosapentaenoic acid (EPA) and docohexaenoic acid (DHA) of the ω-3 family. EPA and DHA have been reported to support and alleviate cardiovascular diseases and inflammatory conditions, and are also the important additives in infant feed formula. Traditionally, ω-3 fatty acids have been obtained from marine fatty fish, and in view of the declining fish sources and the issues of heavy metal contamination of marine fish, these fatty acids can also be extracted from microalgae. Species of the genera *Nannochloropsis*, *Phaeodactylum*, *Schizochytrium* and *Thraustochytrium* have been known to produce ω-3 fatty acids in autotrophic or heterotrophic mode [125]. Microalgal extracts have also been used in the cosmetic industry due to their antioxidant and nourishing properties that combat aging. The antioxidant properties of pigments with nourishing proteins and lipids make them the ideal topical agents for anti-aging, skin lightening, de-pigmentation and a pimple cure due to the antimicrobial activities. *Chlorella* sp., and *Nannochloropsis atomus* are highly used in cosmetic preparations because of their high lipid and protein contents as well as their antioxidative properties [126]. The cultivation of microalgae is also eco-friendly because they do not require herbicides or pesticides [57], and at the same time, they can generate important co-products such as proteins and residual biomass that can be utilized as feed or fertilizer [24]. Moreover, algal biomass is also being used as feed in the aquaculture industry. The aquaculture industry which produces the commercially important fish relies heavily on microalgae as a food source for juvenile fish. *Nannochloropsis* sp., *Pavlova* sp., *Isochrysis* sp., *Tetraselmis* sp., *Thalassiosira weissflogii*, *Dunaliella* sp. and *Chaetoceros* sp. are some of the well-known algal species cultured routinely for aquaculture feed [127]. Some processes in the biofuel conversion technology will produce waste products in the form of solid charcoal residue, i.e., biochar, which can be used as a fertilizer [128]. Biochar has soil-enhancing properties and it provides a direct nutritional benefit to soil and increases the crop productivity.

## 3. Microalgae Harvesting

Microalgae harvesting is a relatively expensive procedure which accounts for 20%–30% of the overall production costs in algae-based biofuel production [129]. This is because the recovery normally involves one or more solid-liquid segregation steps [12] including flocculation, flotation, centrifugal sedimentation and filtration.

There are several harvesting methods available currently, and each one has its own advantages and disadvantages. Therefore, making a proper choice of harvesting method will have an effect on the economic outcome of the investment. The type of harvesting technique used relies solely on the characteristics of the microalgae, for instance the density, size, and desired output [7]. There are several types of harvesting methods that can be grouped into two main categories: bulk harvesting and thickening.

### 3.1. Bulk Harvesting

This objective of this method is to separate the biomass from the bulk suspension. The technologies under this category include flocculation, flotation and gravity sedimentation. The concentration factors, which can be affected by factors such as the initial biomass concentration and the type of technology used, can be as high as 100–800 times to reach 2%–7% of total solid matter.

#### 3.1.1. Flocculation

For many other methods under the bulk harvesting category, flocculation is a pre-requisite because it introduces cationic polymers that neutralize the naturally occurring negative charge on the cell surface of microalgae, which prevents the aggregation of microalgal cells [130]. An added advantage of this method is that the naturally occurring material such as chitosan can also be used as a bio-flocculant [131]. There are several different variations in the flocculation technique: autoflocculation [132] and chemical coagulation [133]. The latter can be further separated into: coagulation using inorganic coagulants [134], organic flocculants [135], or combined flocculation [136]. However, some of the chemical flocculants required for the process can be costly or toxic, which would then increase the overall production cost or affect the quality of the biofuel [137]. It is not our intention to provide an in-depth review on these technologies, as there is already an abundance of literature [130,138,139,140,141] available on this topic.

#### 3.1.2. Flotation

Flotation, a relatively new method, was originally developed in the mineral industry. The first application of the method in algae removal was reported by Phoochinda et al. in 2003 [142]. In this method, air changes into bubbles through a solid/liquid suspension. The algae cells will attach themselves to the dispersed micro-air bubbles that transport the algae cells to the water surface. This method is free from chemicals [12] and can be more efficient than the sedimentation method in terms of transporting microalgae [143]. The process can also aggregate particles with a small diameter (<0.05 mm) [144]. Moreover, the cost for operating a flotation procedure is greatly reduced compared to the conventional industrial-preferred method, i.e., centrifugation. There are several categories of flotation methods: dissolved air flotation (DAF) [145], dispersed air flotation (DiAF) [146], electroflotation [147], jet flotation [148] and dispersed ozone flotation (DiOF) [149]. Each of these techniques has its own advantages and disadvantages. For instance, DiAF has been found to be more energy efficient compared to DAF [142], while jet flotation has been reported to have a 98% of algal harvesting efficiency and can contribute to a reduced resultant phosphorus content [150]. However, the available literature on the flotation process is only on the small-scale flotation processes.

#### 3.1.3. Gravity Sedimentation

This is the most popular method for algae harvesting, especially in wastewater treatment, due to the large volume of wastewater used and the [151] value of biomass generated. This method uses the basis of Stoke’s law in its operation [26], which means that the cells’ density and size will have an effect on the settling characteristics of the aggregated outcome. It is sometimes used in tandem with other processes such as flocculation to enhance the efficiency of this process. It is suitable only for microalgae with a cell size larger than 70 µm, for example *Spirulina* sp. [117], because the low-density microalgal cells do not settle well using this method [138].

### 3.2. Concentration

The methods under this category are more energy-consuming compared to bulk harvesting methods. The slurry of concentrated microalgae can be obtained via centrifugation, filtration and ultrasonic aggregation.

#### 3.2.1. Centrifugation

This is a popular method for aggregating metabolites due to its fast operation and high efficiency. It is also energy-consuming, and the recovery efficiency, which can exceed 95% [152], relies heavily on the factors such as the settling characteristics of the cells, the slurry residence time in the centrifuge, and the settling depth [130]. The weaknesses of this method include high energy consumption and the requirement for maintenance due to free moving parts [153]. Moreover, the exposure of the particles to the high gravitational and shear forces can cause a significant deterioration to the cells [154].

#### 3.2.2. Ultrasonic Aggregation

Sound can be used to aggregate the microalgae cells. The separation efficiency can be as high as 92%, while achieving a concentration factor of 20 times [153]. This method has previously been applied with success in the medical field [155].

#### 3.2.3. Filtration

For large-size microalgae species, filtration is the most convenient and traditional harvesting method. Normally, the filtration process involves a pressure pump and a filter sheet to filter the microalgae. A study by Mohn et al. [156] on *Coelastrum proboscideum* showed that the method can achieve a concentration factor of 245 times. For microalgae with a relatively smaller size, the filter membrane needs to be changed accordingly (using a micro- or ultra-filtration membrane) [157]. However, the high cost of the membrane filter renders the process less economical compared to the centrifugation method [158]. Moreover, the filtration membranes can, at times, be contaminated and this will affect the quality of the filtered microalgae. In addition, a substantial polarization phenomenon caused by the surface charge of cells can change the characteristics of the cells, the exogenous matter, or even the surface of the filter membrane [142].

#### 3.2.4. Electrophoresis

In the electrophoresis process, an electric field is applied to segregate the algal cells based on the naturally occurring negative charge [159]. This method is very versatile, efficient, safe and cost-effective. The method can be adjusted easily by increasing the electrical power to hasten the segregation process [160].

## 4. Conversion of Microalgae to Biofuel

After the microalgae harvesting, the subsequent process is on the purification and conversion of the aggregated microalgae into biofuel. The aggregated MAB should be processed rapidly after collection to prevent decomposition. The preservation of the harvested MAB can be done easily; the simplest and most cost-effective method of which is drying it under the sun. The drying method, however, requires a longer period and also a vast area [161]. Other popular methods include shelf drying [162], spray drying [163] and drum drying [161]. Spray drying is relatively expensive compared to the other methods, and it has been known to inflict considerable damage to the algae [162]. The temperature the microalgae is being exposed to can also affect the lipid content and yield. Widjaja et al. [55] showed that microalgae *Chlorella vulgaris* dried at 60 °C could retain their TAG content, but at temperatures higher than 60 °C, the TAG content and lipid yield of the microalgae decreased. In order to extract the algal metabolites used for the production of biofuels such as carbohydrates and lipids, a cell disruption step is required. This is because the thick cell walls in microalgae protect the cellular components from being exposed to the solvents used for the extraction of intracellular compounds and metabolites of interest [163], thus affecting the efficiency of the extraction process. After disruption of the cells, algal metabolites such as astaxanthin and ß-carotene can be extracted using solvents [130].

There are several processes that convert MAB into biofuel, and these processes can be grouped into two main categories: thermochemical conversion, which is the thermal degradation of organic components in the microalgae to produce fuel [164], and biochemical conversion, which is the process that includes anaerobic digestion, alcoholic fermentation and photobiological H_2_ production [165].

### 4.1. Thermochemical Processes

#### 4.1.1. Gasification

Gasification enables the production of syngas from various potential feedstocks, including microalgae. In this process, MAB is treated with air at temperatures around 800–1000 °C under low oxygen condition to produce syngas (mixture of CO, H_2_, CO_2_, N_2_ and methane) [166]. The resultant low-calorific-value syngas can be directly used in turbines and engines as fuel or used in chemical conversion processes for the synthesis of methanol [167]. Researchers have studied the feasibility of gasification using several types of microalgae. *Spirulina* sp. has been used to generate methanol by Hirano et al. The *Spirulina* sp. slurry was continuously supplied to a reactor and was partially oxidized at temperatures between 800–1000 °C [167]. The composition of the syngas produced included H_2_, CO, CO_2_, and CH_4_, with C_2_H_4_, N_2_, and O_2_ in trace amount. With an increase in temperature, H_2_ concentration increased and the concentrations of CO, CO_2_, and CH_4_ decreased. It was determined that the optimum temperature that produces the highest yield of methanol is 1000 °C [167]. Gasification is advantageous as it accepts microalgal feed in the slurry form with a moisture content up to 15%, and a moisture content of up to 40% has also been applied [168]. Gasification can occur in the presence or absence of catalysts; with catalysts, the reaction temperature can be lower and generally temperature as high as 1300 °C is needed [168].

#### 4.1.2. Thermochemical Liquefaction

The thermochemical liquefaction process converts MAB into bio-oil at a much lower temperature (around 300–350 °C), but at a higher pressure (5–20 MPa), with 15%–20% of microalgae in the slurry feed, in the presence or absence of a catalyst [169]. The major product of hydrothermal liquefaction is bio-crude oil, which can be in the range of 10%–73%; other products include a gaseous mixture of about 8%–20%, an ash content of 0.2%–0.5%, and a nutrient-rich process liquid which can be recycled as a nutrient source [168]. The process is able to produce energy from wet biomass, but can be relatively costly due to the complicated reactors and fuel feed system [170]. For the production of bio-crude oil, *Botyrococcus braunii* biomass with a high moisture content was subjected to hydrothermal liquefaction at temperature 350 °C and pressure 2 MPa for 60 min. The oil yield was 64% *w*/*w*, which was higher than the oil content of the feed biomass, which was around 50%. A recovery greater than 95% was obtained from hydrothermal liquefaction operated at 300 °C [171]. *Dunaliella tertiolecta* was liquefied at 300 °C and 10 MPa, with an oil yield of about 37% on an organic basis and the oil was reported to have a higher heating value (HHV) of 34.9 MJ·kg^−1^ [172].

#### 4.1.3. Pyrolysis

Pyrolysis is one of the most extensively studied topics and it can be considered as a more mature technology relative to other processes [173,174,175]. The pyrolysis process turns microalgae into biofuel, syngas and biochar, without oxygen at medium temperatures [169]. Solid, liquid and gaseous biofuels can be produced by pyrolysis of microalgal biomas depending upon the process used. Microalgae are a potential feedstock for pyrolysis because the bio-oils produced form microalgae are more stable than those produced from ligno-cellulosic biomass [168]. Different modes of pyrolysis are currently used in the industry, each with its own characteristics. Flash pyrolysis and fast pyrolysis, for instance, use a medium temperature of 500 °C and produce more than 50% of liquid [176]. Slow pyrolysis, on the other hand, requires a lower temperature (400 °C) and produces around 35% of gas, 35% of biochar, and 30% of water [176]. Indeed, the oil yield from this process can be 3.4 times higher than that of phototrophic cultivation [174]. The process can be affected by temperature, and at the optimum temperature, an oil yield as high as 55.3% has been reported (for species *Chlorella prothothecoides*) [173].

#### 4.1.4. Direct Combustion

Dried MAB can also be used in an electric generation plant in the traditional way of direct combustion, to replace fossil fuel. The process is usually done in a furnace at temperatures as high as 800 °C [7]. The limitation of this process is that the energy produced must be applied directly, whether for electricity generation or water heating [177]. However, the simplicity of this process also makes it more advantageous for large-scale operation. The efficiency of the combustion power plant usually ranges from 20%–40% [166]. The inclusion of MAB alongside conventional fuel such as coal has shown to produce less GHG emissions [178].

### 4.2. Biochemical Conversion

Biochemical conversion of microalgae involves microorganisms in the conversion of the complex polymeric substances present in the MAB, such as proteins and carbohydrates, into fuels such as bioethanol, biobutanol, biohydrogen and biomethane (Table 6). Biomethane is the product of the anaerobic digestion of whole or spent MAB, whereas alcoholic fermentation of whole or spent MAB produces ethanol, butanol and H_2_. All these products from MAB can be used for high efficiency combustion in vehicle or power plants. Different types of microalgae species can be used as the feedstock to produce methane, as shown in Table 7. For any biochemical conversion process, pre-treatment of the MAB is necessary. Pre-treatment helps in breaking the rigid cell wall of microalgae and in the efficient release of the cellular components to be used in the subsequent fermentation reactions. Both anaerobic digestion and alcoholic fermentation are discussed in the following sections.

#### 4.2.1. Anaerobic Digestion (AD)

AD is the decaying process of organic matter in the absence of oxygen and other terminal electron acceptors such as sulfate, nitrate or ferric iron, to produce methane and CO_2_. Therefore, microalgae can be used as the organic matter for AD conversion into usable biomethane [180]. It is a complex process involving a consortium of anaerobic bacteria, and the reactions between the substrate and the host are multifold. AD is a widely accepted method for the treatment of solid sewage, the organic fraction of municipal sewage and the digestion of manure. In microalgae processing, AD can be used to transform the entire MAB into biogas in a single step [181]. AD occurs in four stages: hydrolysis, acidogenesis, acetogenesis and methanogenesis, with the hydrolysis step being the rate-limiting step in AD [182].

The hydrolysis step involves the degradation of insoluble organic material and high-molecular-weight compounds such as lipids, polysaccharides, proteins and nucleic acids into soluble organic compounds such as monosaccharides or amino acids. In the next step (acidogenesis), these resultant compounds are further degraded to produce volatile fatty acids (VFA), ammonia and CO_2_. After going through acidogenesis, the products from the acidogenesis step (i.e., organic acids and alcohols) are digested by acetogens into acetic acid, CO_2_ and H_2_ in a process called acetogenesis, which is affected by the partial pressure of the H_2_ in the mixture. Lastly, products from acetogenesis are converted into the usable methane by using two groups of methanogenic bacteria: the first group transforms acetate into methane and CO_2_, while the second group converts H_2_ and CO_2_ into methane [171].

As mentioned above, hydrolysis is one of the main rate-limiting steps in AD, and it is heavily influenced by the type of cell wall structure of the microalgae. The cell wall structures of microalgae vary widely, and, generally, marine microalgae have a thicker cell wall compared to fresh water microalgae. The presence of a strong cell wall is challenging as it cannot be broken down easily and this ultimately affects the hydrolysis step, and thereby the overall efficiency of AD. Thanks to its less complex cell wall, freshwater microalgae require only a mild treatment for AD.

The presence of salt such as ammonium can also inhibit the efficiency of AD. Ammonium exists in two forms: the protonated form (NH_4_^+^) and the deprotonated form (NH_3_). Deprotonated ammonium inhibits AD because of its permeability through microalgal cell walls. The distribution of both forms of ammonium is affected by the pH and temperature of the culture system. A higher pH encourages the production of NH_3_, while lowering the methanogenic activity reduces the production of NH_3_, resulting in a drop in pH. The concentrations of inhibitors that can affect AD are summarized in Table 8. Temperature affects AD performance by changing the physical and physiochemical properties of the medium and the thermodynamics of biological processes. The mesophilic condition is described as a moderate temperature (30–38 °C) and this condition is suitable for the growth of mesophiles, while the thermophilic condition (49–57 °C) is described as a warmer temperature [183,184]. In comparison to mesophilic condition, the thermophilic condition is more ideal for AD, as it provides a better waste stabilization, a more sludge dewatering, a higher production of methane, a greater hydrolysis rate, a lower formation of foam and a better reduction of volatile organics. It also has a high operating coss due to the higher energy consumption, the longer period of sludge adaptation, the low stability, the vulnerability to various chemicals such as ammonium, potassium or sodium inhibition, and the high generation of volatile fatty acids that could affect the pH. Nutrients such as phosphorus and ammonium which, in the form phosphate and ammonia, respectively, are produced during AD [185], and they can be reused as a substrate for microalgae cultivation.

#### 4.2.2. Alcoholic Fermentation

Alcoholic fermentation for the production of biofuels, such as ethanol, butanol and H_2_, involves the conversion of the carbohydrates present in the MAB to alcohols by the action of various microorganisms. Bacteria, yeast and fungi are the commonly used microorganisms to ferment the carbohydrates in the microalgae into ethanol and CO_2_ under anaerobic condition. The CO_2_ produced from the fermentation process can be recycled into the algae cultivation ponds for the growth of microalgae. A simplified fermentation equation is shown in Equation (1).

(1) $$C_{6}H_{12}O_{6}\to {2C}_{2}H_{6}O+{\mathrm{CO}}_{2}$$

However, the complex cell wall structure of microalgae can be a deterrent for the microorganisms to reach the carbohydrates. Therefore, pre-treatment is necessary for the efficient release of carbohydrates and their hydrolysis into monomers for uptake and conversion by microorganisms. Various pre-treatment methods have been employed by researchers to efficiently release the microalgal carbohydrates: physical or mechanical, thermal, chemical and enzymatic methods. Of these, the most commonly used for the release of microalgal sugars is a combined thermal and chemical pre-treatment processes, which involve the hydrolysis of MAB in the presence of mild acid/alkali at elevated temperature. Since microalgae are devoid of lignin, this simple pre-treatment often gives a high recovery of simple sugars with a greater efficiency [186].

In general, species of the genera *Chlorella*, *Dunaliella*, *Chlamydomonas*, *Scenedesmus*, *Tetraselmis* and *Spirulina* are known to accumulate a higher content of carbohydrates (>40% by weight) under certain nutrient deprivation conditions, such as nitrogen and phosphorus [187]. The carbohydrates of microalgae are either associated with their cells as structural polysaccharides such as cellulose and hemicellulose, or as storage polysaccharides which are mostly starch or glycogen in green algae and cyanobacteria. Hydrolysis of these carbohydrates yields glucose and xylose as the major sugars, and other sugars such as mannose, arabinose and galactose. All these sugars can be efficiently utilized by the fermenting microorganisms [187].

The most commonly used ethanologen for the industrial production of ethanol is the yeast *Saccharomyces cerevisiae*. *S. cerevisisae* utilizes most of the hexoses and it converts them to ethanol, but it is incapable of utilizing pentoses, which are most commonly present in the feedstocks used for bioethanol production, including hydrolysates of microalgae or lignocellulosic biomass. In addition, the invariable presence of a higher salt concentration in the hydrolysate of marine microalgae can affect the ethanol fermentation efficiency of *S. cerevisiae*. Markou et al. utilized the carbohydrate-rich biomass of *Arthrospira platensis* (60% by weight of carbohydrate) for ethanol fermentation with a salt stress–adapted *S. cerevisiae*. A combination of acid and thermal treatments was used and it was observed that an increase in both the acid strength and temperature improved the recovery of the reducing sugars, while using low-strength acids at a higher temperature yielded the optimal results. The highest bioethanol yield of around 16.5% was achieved for both 0.5 N sulphuric acid- and nitric acid-treated *A. platensis* biomass [188]. The carbohydrate-rich biomass of *Chlorella vulgaris* FSP-E (51% carbohydrate) was pretreated by acid and enzymatic methods, and was used for ethanol fermentation using the alternative ethanologen *Zymomonas mobilis* [189]. *Z. mobilis* is known for its higher sugar uptake and ethanol yield, lower biomass production, higher ethanol tolerance up to 120 g/L, lack of controlled addition of oxygen during fermentation, and high possibility for genetic manipulation. Enzymatic hydrolysis of *C. vulgaris* FSP-E gave a glucose yield of about 90.4%, while mild acid hydrolysis achieved a glucose yield of 93.6%. However, Super high frequency (SHF) with enzymatic hydrolysate yielded about 79.9% of the theoretical yield, whereas SHF with acid hydrolysate achieved 87.6% of the theoretical yield [189].

Biobutanol, the only drop-in liquid fuel to be used with gasoline, can also be produced by clostridial acetone-butanol-ethanol fermentation using microalgal hydrolysate as a feedstock. In acetone–butanol–ethanol (ABE) fermentation, H_2_ is also obtained as a byproduct, and thus the production of both liquid and gaseous biofuels can be achieved in a single step. Castro et al. utilized wastewater algae for the production of biobutanol by *Clostridium saccharoperbutylacetonicum* [190]. The wastewater microalgae consist mainly of *Scenedesmus*, *Chlorella*, *Ankistrosdemus*, *Micromonas*, and *Chlamydomonas* species. The pre-treatment with 1.0 M sulphuric acid at 80–90 °C for 120 min was found to be optimal for the acid hydrolysis, and a reducing sugar yield of 166.1 g per kg of dry algae was achieved. ABE fermentation with 10% of pre-treated algae achieved 5.23 g/L of total ABE and 3.74 g/L of butanol at a price of USD 12.54 per kg of butanol [190]. SMAB obtained after lipid extraction for biodiesel conversion can also be used as a substrate for ABE fermentation and butanol production. SMAB of *Chlorella sorokiniana* CY1 after lipid extraction using methanol and hexane was subjected to mild acid hydrolysis and was used in ABE fermentation by *C. acetobutylicum* [191]. A butanol yield of 3.86 g/L was achieved when microalgal sugars were used at a concentration of 300 g/L, and a high yield of 0.13 g/g-carbohydrate was obtained with 100 g/L of microalgal sugars [191].

Microalgal carbohydrates have also been used as a substrate for dark fermentative H_2_ production. The carbohydrate-rich biomass or the spent microalgae from biodiesel production can be used after appropriate pre-treatment. *Chlorella vulgaris* FSP-E was grown in mixotrophic mode using sodium acetate as the organic carbon source, and an enhanced biomass and carbohydrate productivity of 1022.3 mg/L/day and 498.5 mg/L/day, respectively, were achieved. The carbohydrate-rich biomass (54.84%) was pretreated by the mild acid-thermal method, and the resultant hydrolysate was used as a feedstock for H_2_ fermentation using *Clostridium butyricum* CGS5 [192]. A H_2_ production and yield of 176.9 mL/h/L and 2.87 mmol H_2_/g biomass were obtained, respectively [193]. SMAB can also be valorized via the dark fermentative H_2_ production. *Scenedesmus* sp. biomass derived from the oil extraction process with a carbohydrate content of 24.7% was used in H_2_ production with heat-treated anaerobic sludge. The optimum conditions were found to be 36 g/L volatile solids loading, an initial pH of 6.0–6.5 and the heat treatment of sludge and acetate; propionate and butyrate were obtained as main the end products along with H_2_ [194].

#### 4.2.3. Photobiological H_2_ Production

H_2_ can be produced by using MAB as a feedstock in dark fermentative fermentation by anaerobic bacteria as discussed in the previous section. However, H_2_ gas can also be produced directly by microalgae by the water-splitting activity of the photosystems involved in photosynthesis. In these light-dependent systems, the water-splitting activity of photosystem II (PSII) is supplied with electrons by the light-excited photosystem I (PSI) or by the intracellular plastoquinone pool derived from the metabolism of intracellular carbohydrates. Either way, photosystem II is the water-splitting component and the resultant electrons are transferred to protons to produce H_2_, catalyzed by the enzyme hydrogenase [195]. Hydrogenase is widely distributed in green algae and cyanobacteria. The hydrogenases of green algae can only generate H_2_, while the hydrogenases of cyanobacteria are bidirectional and can utilize H_2_ that has been released during N_2_ fixation. H_2_ production by green algae and cyanobacteria is temporary, occurring only under certain associated physiological conditions such as sudden illumination and oxygen deprivation in green algae or N_2_ fixation in cyanobacteria [195]. A continuous H_2_ production has been achieved in green algae, particularly in *Chlamydomonas reinhardtii* using a two-phase approach involving macronutrient deprivation. Sulfur deprivation of the microalgal cultures after exponential growth resulted in a partial inactivation of PSII, lowering its activity, inducing cellular respiration, establishing anoxia and attaining sustained H_2_ production for a period of five to seven days [196]. Even though this strategy works well, it is also transient and the resultant MAB needs to be taken care of. Despite the many advantages of using microalgae for photobiological H_2_ production, commercialization of this approach is a long-term objective and needs more inputs in terms of operation feasibility on an industrial scale.

## 5. Converting MAB to Biodiesel

Apart from producing other liquid biofuels, microalgae are mostly exploited for the production of biodiesel [197]. Currently, biodiesel is already being produced commercially using products from animal fat, used edible oil and vegetable oil [198]. Corn starch, sugar cane or sugar beets are used to produce bioethanol while palm and oilseed rape are harvested for the production of biodiesel [177]. With the increasing demand for biodiesel, industries are turning to microalgae as a new biodiesel source, as the fatty acid profile extracted from microalgal oil is compatible with the production of biodiesel [199]. After drying and processing, the microalgal oil or lipids are extracted for transesterification. Transesterification, also called alcoholysis, is the displacement of alcohol from an ester by another alcohol in a process similar to hydrolysis, except that an alcohol is employed instead of water [200]. An alcohol such methanol, ethanol, propanol, butanol and amyl alcohol can be used for alcoholysis, while ethanol and methanol are most frequently used. If methanol is used, the process is called methanolysis. Transesterification can happen in the presence or absence of catalysts, and the catalysts can be chemical or biological agents (enzymes). The product of the transesterification of fatty acid methyl esters (FAME) can be used as a fuel in diesel engines [200]. Table 9 shows the biodiesel from different sources with the yield [201]. An advantage of using microalgae to produce biodiesel is the high yield: up to 58,700 L of oil can be synthesized from every hectare of microalgae, making it at least a magnitude or two greater than the oil produced from other crops [82] (Table 10).

## 6. Prospects and Challenges

Microalgal cultivation for the production of biofuels can be more meaningful and cost-competitive if the biofuel production process is integrated with the extraction of other valuable biomolecules from the biomass. Normally, lipid-extracted microalgal biomass (MAB), also known as spent microalgal biomass (SMAB), is a potential source of various bioactive compounds. The term microalgal biorefinery [202] is applied to such approaches where the MAB is utilized for the production of a number of valuable products including biofuels, fine chemicals, bioactive compounds and so on [203]. The carbohydrates and lipids from microalgae have been established as an efficient feedstock for the production of biofuels, while other constituents such as fatty acids, proteins and antioxidants can be used as pharmaceuticals and nutraceuticals. The resultant biomass after the extraction process can be further treated by AD or used as a sorbent in bioremediation for pollutant removal in wastewaters [204]. Moreover, MAB can be cultivated using the nutrient-rich wastewater and the industrial exhaust gases as a source of CO_2_, thereby cutting production costs. The considerable enhancements in pretreatment processes are imperative for the restructuring of macromolecules (e.g., proteins) while improving MAB digestibility. The efficiency of the biomass relies heavily upon upstream processes. More research effects need to be directed towards integrating the upstream and downstream processes of biomass slurry for a more effective and efficient energy recovery.

Nutrient losses can be mitigated by adjusting the parameters and the process conditions for nutrient recycling. If the technical challenges on the recycling of algae nutrients can be tackled, the ability to recycle nutrients could be one of the great advantages of microalgae farming over the conventional algae farming [61]. Hence, it is imperative to develop suitable and sustainable treatment technologies that can recycle the nutrients from the residues of algae biofuel processing for use in algae growth.

A techno-economics analysis should be conducted to set a proper goal or future objective for microalgae-based biofuels. As discussed above, MAB can be used in the production of biodiesel and bioethanol. In the future, however, the application of microalgae must be diversified and extended to include bioremediation alongside biofuel generation [184]. The commercial initiatives for this rely on: the constituents and the volume of the effluent, the microalgae species, as well as the temperature and light conditions [205]. The initiatives will also depend on the particular biofuel of interest for the regional or local consumption [206]. Hence, every circumstance should be analyzed individually, and it must be noted that there is no one single model that fits all the requirements.

The sustainability of MAB also depends on the operational costs and the environmental impacts of the process. The reuse and recycling the MAB are known to be cost effective. It is difficult to justify the time and money invested in MAB if the return extracted from biomass technology is low and insignificant. Hence, microalgal biorefinery approaches make more sense when it comes to sustainable fuel production from MAB.

## Figures and Tables

**Table 1:** Yields of biofuel from different crops. Used with permission from [14].

Crops	Gallon acre^−1^·year^−1^
Corn	15
Soybean	48
Sunflower	102
Rapeseed	127
Oil palm	635
Microalgae—actual biomass yield	1850
Microalgae—theoretical laboratory yield	5000–15,000

**Table 2:** Lipid content from different microalgae species. Modified from [14] with added information from [39].

Microalgae Species	Lipid Content (% Dry Weight Biomass)	Lipid Productivity (mg/L/day)
*Ankistrodesmus* sp.	24.0–31.0	-
*Botryococcus braunii*	25.0–75.0	-
*Chaetoceros muelleri*	33.6	21.8
*Chaetoceros calcitrans*	14.6–16.4/39.8	17.6
*Chlorella emersonii*	25.0–63.0	10.3–50.0
*Chlorella protothecoides*	14.6–57.8	1214
*Chlorella sorokiniana*	19.0–22.0	44.7
*Chlorella vulgaris*	5.0–58.0	11.2–40.0
*Chlorella* sp.	10.0–48.0	42.1
*Chlorella pyrenoidosa*	2.0	-
*Chlorella*	18.0–57.0	18.7
*Chlorella zofingiensis*	45.5	473.0
*Chlorococcum* sp.	19.3	53.7
*Crypthecodinium cohnii*	20.0–51.1	-
*Dunaliella salina*	6.0–25.0	116.0
*Dunaliella primolecta*	23.1	-
*Dunaliella tertiolecta*	16.7–71.0	-
*Dunaliella* sp.	17.5–67.0	33.5
*Ellipsoidion* sp.	27.4	47.3
*Euglena gracilis*	14.0–20.0	-
*Haematococcus pluvialis*	25.0	-
*Isochrysis galbana*	7.0–40.0	-
*Isochrysis* sp.	7.1–33	37.8
*Monodus subterraneus*	16.0	30.4
*Monallanthus salina*	20.0–22.0	-
*Nannochloris* sp.	20.0–56.0	60.9–76.5
*Nannochloropsis oculata*	22.7–29.7	84.0–142.0
*Nannochloropsis* sp.	12.0–53.0	37.6–90.0
*Neochloris oleoabundans*	29.0–65.0	90.0–134.0
*Nitzschia* sp.	16.0–47.0	-
*Oocystis pusilla*	10.5	-
*Pavlova salina*	30.9	49.4
*Pavlova lutheri*	35.5	40.2
*Phaeodactylum tricornutum*	18.0–57.0	44.8
*Porphyridium cruentum*	9.0–18.8/60.7	34.8
*Scenedesmus obliquus*	11.0–55.0	-
*Scenedesmus quadricauda*	1.9–18.4	35.1
*Scenedesmus* sp.	19.6–21.1	40.8–53.9
*Skeletonema* sp.	13.3–31.8	27.3
*Skeletonema costatum*	13.5–51.3	17.4
*Spirulina platensis*	4.0–16.6	-
*Spirulina maxima*	4.0–9.0	-
*Thalassiosira pseudonana*	20.6	17.4
*Tetraselmis suecica*	8.5–23.0	27.0–36.4
*Tetraselmis* sp.	12.6–14.7	43.4

**Table 3:** Maximum productivities of biomass and key components for *Chlorella zofingiensis*. Adapted from [39].

Growth Condition	Maximum Productivities		
	Biomass (g·L^−1^·day^−1^)	Triacylglycerol (mg·L^−1^·day^−1^)	Astaxanthin (mg·L^−1^·day^−1^)
Low Light	0.83 ± 0.05	11.3 ± 0.7	0.05 ± 0.01
Nitrogen Deprivation	0.41 ± 0.02	91.5 ± 5.5	1.08 ± 0.06
High Light	1.40 ± 0.09	173.6 ± 11.2	2.01 ± 0.14
Nitrogen Deprivation + High Light	0.53 ± 0.03	145.8 ± 9.7	1.79 ± 0.17

**Table 4:** Biomass productivity figures for open pond production system. Adapted from [7].

Algae Species	X_max_ (g·L^−1^)	P_aerial_ (g·m^−2^·day^−1^)	P_volume_ (g·L^−1^·day^−1^)	PE (%)
*Chlorella* sp.	10	25	-	-
N/A	0.14	35	0.117	-
*Spirulina platensis*	-	-	0.18	-
*Spirulina platensis*	0.47	14	0.05	-
*Haematococcus pluvialis*	0.202	15.1	-	-
*Spirulina*	1.24	69.16	-	-
*Spirulina platensis*	0.9	12.2	0.15	-
*Spirulina platensis*	1.6	19.4	0.32	-
*Anabaena* sp.	0.23	23.5	0.24	>2
*Chlorella* sp.	40	23.5	-	6.48
*Chlorella* sp.	40	11.1	-	5.98
*Chlorella* sp.	40	32.2	-	5.42
*Chlorella* sp.	40	18.1	-	6.07

**Table 5:** CO_2_ and biomass productivity for CO_2_ mitigation species. Adapted from [7].

Microalgae	T (°C)	CO_2_ (%)	P_volume_ (g·L^−1^·day^−1^)	$P_{co_{2}}$ (g·L^−1^·day^−1^)	Carbon Usage Efficiency (%)
*Chlorella* sp.	26	Air	0.682 ^a^	-	-
*Chlorella* sp.	26	2	1.445 ^a^	-	58
*Chlorella* sp.	26	5	0.899 ^a^	-	27
*Chlorella* sp.	26	10	0.106 ^a^	-	20
*Chlorella* sp.	26	15	0.099 ^a^	-	16
*Chlorella kessleri*	30	18	0.087	-	-
*Scenedesmus* sp.	25	10	0.218	-	-
*Chlorella vulgaris*	25	10	0.105	-	-
*Botryococcus braunii*	25	10	0.027	-	-
*Scenedesmus* sp.	25	Flue gas	0.203	-	-
*Botryococcus braunii*	25	Flue gas	0.077	-	-
*Chlorella vulgaris*	25	Air	0.040	-	-
*Chlorella vulgaris*	25	Air	0.024	-	-
*Haematococcus pluvialis*	20	16–34	0.076	0.143	-
*Scenedesmus obliquus*	-	Air	0.009	0.016	-
*Scenedesmus obliquus*	-	Air	0.016	0.031	-
*Chlorella vulgaris*	27	15	-	0.624	-
*Scenedesmus obliquus*	30	18	0.14	0.260	-
*Spirulina* sp.	30	12	0.22	0.413	-

^a^ Culture incubated for four to eight days.

**Table 6:** Properties of biobutanol and bioethanol as vehicular fuel. Adapted from [179].

Properties	Butanol	Ethanol
Melting point (°C)	−89.3	−114.0
Specific gravity	0.810–0.812	0.79
Ignition temperature (°C)	35–37	276–456
Auto-ignition temperature (°C)	343–345	422
Flash point (°C)	25–29	12.77
Relative density	0.81	0.805–0.812
Critical temperature (°C)	287	239.85
Explosive limits (vol % in air)	1.4–11.3	3.3–19.0
Vapor pressure (kPa at 20 °C)	0.5	5.95
Boiling point (°C)	117–118	78
Density at 20 °C (g/mL)	0.8098	0.7851
Energy density (MJ/L^−1^)	27.0–29.2	19.6
Energy content (BTU/gal)	110,000	84,000
Liquid heat capacity at STP (kJ/kmol·°K)	178	112.3
Research octane number	96	129
Motor octane number	78	102
Viscosity (10^−3^ Pa·s)	2.593	1.078

**Table 7:** Yield of methane from various feedstocks. Used with permission from [179].

Biomass	Methane Yield (m^3^·kg^−1^)
*Laminaria* sp.	0.26–0.28
*Gracilaria* sp.	0.28–0.40
*Sargassum* sp.	0.12–0.19
Macrocystis	0.39–0.41
*L. digitata*	0.50
*Ulva* sp.	0.20
Water hyacinth	0.13–0.21
Sorghum	0.26–0.39
Poplar	0.23–0.32
Food waste	0.54
Microalgae—ACAD model	0.54

**Table 8:** The concentration of inhibitors that can affect AD [14].

Inhibitor	Moderate Inhibitory Concentration (mg·L^−1^)	Strongly Inhibitory Concentration (mg·L^−1^)
Na^+^	3500–5500	8000
NH_4_^+^	1500–3500	3000
K^+^	2500–4500	12,000
Ca^2+^	2500–4000	8000
Mg^2+^	1000–1500	3000
S^2−^	200	200
Cu^2+^	ns ^(1)^	0.5 ^(2)^
Cr^3+^	ns ^(1)^	200–250 ^(3)^
Cr^6+^	10	3.0 ^(2)^
Zn^2+^	ns ^(1)^	1.0 ^(2)^
Ni^2+^	ns ^(1)^	30 ^(3)^
VFAs	ns ^(1)^	6.7–9.0 ^(4)^
18-C LCFA	ns ^(1)^	1000

^(1)^ Not specified in the bibliography; ^(2)^ soluble; ^(3)^ total; ^(4)^ value in mol m^−3^.

**Table 9:** Comparison of microalgae with other biodiesel feedstocks. Adapted from [14].

Plant Source	Seed Oil Content (% Oil by wt in Biomass)	Oil Yield (L·Oil/ha-Year)	Land Use (m^2^·Year/kg Biodiesel)	Biodiesel Productivity (kg·Biodiesel/ha-Year)
Corn (*Zea mays* L.)	44	172	66	152
Hemp (*Cannabis sativa* L.)	33	363	31	321
Soybean (*Glycine max* L.)	18	636	18	562
Jatropha (*Jatropha curcas* L.)	28	741	15	656
Camelina (*Camelina sativa* L.)	42	915	12	809
Rapeseed (*Brassica napus* L.)	41	974	12	862
Sunflower (*Helianthus annuus* L.)	40	1070	11	946
Castor (*Ricinus communis*)	48	1307	9	1156
Palm oil (*Elaeis guineensis*)	36	5366	2	4747
Microalgae (low oil content)	30	58,700	0.2	51,927
Microalgae (medium oil content)	50	97,800	0.1	86,515
Microalgae (high oil content)	70	136,900	0.1	121,104

**Table 10:** Comparison of open ponds, PBRs and fermenters. Used with permission from [201].

Parameter	Open Pond	PBR	Fermenter
Land requirement	High	Varied	Low
Water loss	Very high	Low	Low
Hydrodynamic stress on algae	Very low	Low-high	Unknown
Gas transfer control	Low	High	High
CO_2_ loss	High	Low	No CO_2_ required
O_2_ inhibition	Usually low enough due to continuous spontaneous outgassing	High	O_2_ supply should be sufficient
Temperature	Highly varied	Cooling required	Needs to be maintained
Startup period	6–8 weeks	2–4 weeks	2–4 weeks
Construction costs	USD $100,000 per hectare	USD $1 million per hectare	Low
Operation costs	Low	Very high	Very high
Limiting factor for growth	Light	Light	O_2_
Control over parameters	Low	Medium	Very high
Technology	Readily available	Under development	Readily available
Pollution risk	High	Medium	Low
Pollution control	Difficult	Easy	Easy
Species control	Difficult	Easy	Easy
Weather dependence	High: light intensity, temperature, rainfall	Medium	Low
Maintenance	Easy	Difficult	Difficult
Cleaning	Easy	Difficult	Difficult
Overheating risk	Low	High	Unknown
Excessive O_2_ levels risk	Low	High	Unknown
Cell density in culture	0.1–0.5 g·L^−1^	2–8 g·L^−1^	15.5 or even 80.0–110.0 g·L^−1^
Light-induced products (pigments, chlorophyll, etc.)	No impact	No impact	Reduced
Surface area-to-volume ratio	High	Very high	Not applicable
Applicability to different species	Low	High	Low
Ease of scale-up	High	Varied	High
